# *GmWRKY16* Enhances Drought and Salt Tolerance Through an ABA-Mediated Pathway in *Arabidopsis thaliana*

**DOI:** 10.3389/fpls.2018.01979

**Published:** 2019-01-21

**Authors:** Qibin Ma, Zhenglin Xia, Zhandong Cai, Lu Li, Yanbo Cheng, Jia Liu, Hai Nian

**Affiliations:** ^1^The State Key Laboratory for Conservation and Utilization of Subtropical Agro-Bioresources, South China Agricultural University, Guangzhou, China; ^2^The Key Laboratory of Plant Molecular Breeding of Guangdong Province, College of Agriculture, South China Agricultural University, Guangzhou, China; ^3^National Engineering Research Center of Plant Space Breeding, South China Agricultural University, Guangzhou, China; ^4^The Guangdong Subcenter of the National Center for Soybean Improvement, College of Agriculture, South China Agricultural University, Guangzhou, China

**Keywords:** *GmWRKY16*, soybean, salt, drought, ABA, *Arabidopsis thaliana*

## Abstract

The WRKY transcription factors (TFs) are one of the largest families of TFs in plants and play multiple roles in plant development and stress response. In the present study, *GmWRKY16* encoding a WRKY transcription factor in soybean was functionally characterized in Arabidopsis. GmWRKY16 is a nuclear protein that contains a highly conserved WRKY domain and a C_2_H_2_ zinc-finger structure, and has the characteristics of transcriptional activation ability, presenting a constitutive expression pattern with relative expression levels of over fourfold in the old leaves, flowers, seeds and roots of soybean. The results of quantitative real time polymerase chain reaction (qRT-PCR) showed that *GmWRKY16* could be induced by salt, alkali, ABA, drought and PEG-6000. As compared with the control, overexpression of *GmWRKY16* in Arabidopsis increased the seed germination rate and root growth of seedlings in transgenic lines under higher concentrations of mannitol, NaCl and ABA. In the meantime, *GmWRKY16* transgenic lines showed over 75% survival rate after rehydration and enhanced Arabidopsis tolerance to salt and drought with higher proline and lower MDA accumulation, less water loss of the detached leaves, and accumulated more endogenous ABA than the control under stress conditions. Further studies showed that *AtWRKY8*, *KIN1*, and *RD29A* were induced in *GmWRKY16* transgenic plants under NaCl treatment. The expressions of the ABA biosynthesis gene (*NCED3*), signaling genes (*ABI1*, *ABI2*, *ABI4*, and *ABI5*), responsive genes (*RD29A*, *COR15A*, *COR15B*, and *RD22*) and stress-related marker genes (*KIN1, LEA14, LEA76*, and *CER3*) were regulated in transgenic lines under drought stress. In summary, these results suggest that *GmWRKY16* as a WRKY TF may promote tolerance to drought and salt stresses through an ABA-mediated pathway.

## Introduction

The growth and yield of plants are seriously affected and inhibited by various biotic and abiotic stresses such as high salinity, drought and extreme temperatures, which are the main limiting factors (Hennig, 2012; Liang et al., 2017; He et al., 2018). Plants have evolved some effective mechanisms to face environmental challenges (Munns, 2002; He et al., 2018). Among all the stress-resistance systems in plants, many transcription factors (TFs) related to plant tolerance to stresses have been identified as playing important roles in abiotic stresses. Under stressful conditions, transcriptome changes are the earliest responses in plants. The TFs from the bZIP, NAC, AP2, WRKY, PHD, DREB, or MYB families are essential for plant stress response by binding specific *cis*-acting elements to form a complex regulatory network. Overexpression of these TF genes usually increases the adaptability of plants to drought and salt stresses (Liu et al., 2012; Ying et al., 2012; Zong et al., 2016; Ullah et al., 2018).

The WRKY TFs, dominating the genetic transcription, have become one of the largest TF families in plants (Eulgem et al., 2000; Rushton et al., 2010; Bencke-Malato et al., 2016; Goel et al., 2016; Meng et al., 2016; Yu Y. et al., 2016; Wu J. et al., 2017; Xiao et al., 2017; Bai et al., 2018; Finatto et al., 2018; Song et al., 2018; Xie et al., 2018). All the WRKY proteins have one or two conserved WRKY domains of approximately 60 amino acids with a highly conserved sequence motif WRKYGQK and a Zn-finger motif at N and C termini, respectively (Eulgem et al., 2000). The WRKY TFs can be classified into three groups (I, II, and III) according to the number of WRKY domains and the Zn-finger motif type. The WRKY proteins of group I have two domains of WRKY and a C_2_H_2_-type zinc-finger structure, while the WRKY proteins of groups II and III have a WRKY domain with C_2_H_2_ and/or C_2_HC-type Zn-finger motifs, respectively. Most TF proteins of group II with one WRKY domain are divided into five subgroups (IIa–IIe) on their phylogenetic clades (Eulgem et al., 2000; Zhang and Wang, 2005; Rushton et al., 2010; Zhang et al., 2017; Song et al., 2018; Xie et al., 2018; Yang et al., 2018).

Since the first WRKY gene *SPF1* was isolated from sweet potato (Ishiguro and Nakamura, 1994), more WRKY family genes with multiple roles in plants were discovered and widely reported to be particularly involved in response to biotic and abiotic stresses (Dai et al., 2016; Kiranmai et al., 2018) as well as regulatory roles such as leaf senescence (Miao and Zentgraf, 2007; Rinerson et al., 2015), trichome development (Bakshi and Oelmüller, 2014), root growth (Grunewald et al., 2013), seed development (Schluttenhofer and Yuan, 2015), dormancy (Ding et al., 2014), and secondary metabolites biosynthesis (Eulgem et al., 2000).

Recently, the functional characterization of WRKY proteins in plants was focused on their response to abiotic stresses. An individual WRKY gene can enhance plant tolerance to abiotic stress (Wang et al., 2013; Bakshi and Oelmüller, 2014; Dai et al., 2016; Kiranmai et al., 2018). In rice, overexpression of *OsWRKY11* and *OsWRKY42* improved heat and/or salt tolerance in transgenic rice seedlings (Wu et al., 2009; Pillai et al., 2018), and overexpression of *OsWRKY11* and *OsWRKY30* enhanced drought tolerance in transgenic rice (Wu et al., 2009; Shen et al., 2012), whereas *OsWRKY76* played opposite roles in cold stress tolerance and blast disease resistance (Yokotani et al., 2013). In Arabidopsis, overexpression of *AtWRKY25*, *AtWRKY26* and *AtWRKY30* enhanced resistance to heat and salt stresses, respectively (Li et al., 2011; Scarpeci et al., 2013); overexpression of *AtWRKY46*, *AtWRKY54*, *AtWRKY70*, and *ABO3* enhanced resistance to drought stress (Ren et al., 2010; Chen et al., 2017). In contrast to rice and Arabidopsis, many WRKY genes in other plants have been identified specifically in relation to abiotic stress. For example, *GhWRKY41/SpWRKY1* enhances salt and drought tolerance in transgenic tobacco by regulating stomatal conductance and ROS levels (Li et al., 2015; Chu et al., 2016). Other WRKY genes such as *TaWRKY2* and *TaWRKY19* (wheat), *TaWRKY146* (wheat), *TaWRKY1* and *TaWRKY33* (wheat), *VaWRKY14* (grapevine), *ZmWRKY17* and *ZmWRKY40* (maize), and *PeWRKY83* (moso bamboo) are overexpressed in Arabidopsis, and the heterologous transgenic plants of the WRKY genes conferred tolerance to drought and/or salt stresses, respectively (Niu et al., 2012; He et al., 2016; Cai et al., 2017; Ma et al., 2017; Wu M. et al., 2017; Wang et al., 2018; Zhang et al., 2018). Among the WRKY genes in soybean, the overexpression of *GmWRKY21* enhanced the tolerance to cold stress in Arabidopsis, whereas *GmWRKY54* conferred salt and drought tolerance. By contrast, overexpression of *GmWRKY13* resulted in more sensitivity to salt and mannitol stress (Zhou et al., 2008). *GmWRKY27* overexpression improved salt and drought tolerance by interacting with *GmMYB174* to reduce expression of *GmNAC29* (Wang et al., 2015).

The plant hormone abscisic acid (ABA) plays important roles in the regulation of plant responses to adverse environmental stresses including drought, extreme temperatures and osmotic stress partly mediated by ABA-independent and -dependent pathways (Shinozaki and Yamaguchi-Shinozaki, 1997; Fujii and Zhu, 2009; Umezawa et al., 2010; Rushton et al., 2012; Shang et al., 2012; Yoshida et al., 2014; Song et al., 2016). Recently, some WRKY genes responding to abiotic stresses have been reported within the ABA signaling pathways. *GhWRKY6*-like from cotton improved Arabidopsis tolerance to salt stress by activating the ABA signaling pathway and eliminating reactive oxygen species (Ullah et al., 2018). *GhWRKY17* in transgenic nicotiana enhanced the sensitivity of plants to salt and drought stresses through the ABA signaling pathway by reducing the level of ABA and transcript levels of some ABA-inducible genes (Yan et al., 2014). The overexpression of *ZmWRKY17* in Arabidopsis resulted in the enhanced sensitivity of transgenic plants to salt stress and decreased ABA sensitivity by regulating the expression of some ABA- and stress-responsive genes (Cai et al., 2017). *GsWRKY20* and *ABO3* in Arabidopsis increased drought tolerance of transgenic plants by regulating gene expression of the ABA signaling pathway (Ren et al., 2010; Luo et al., 2013). *CmWRKY1* and *CmWRKY10* enhanced drought and dehydration tolerance of chrysanthemum by an ABA-mediated pathway through the regulation of ABA-associated genes (Fan et al., 2016; Jaffar et al., 2016).

In this study, *GmWRKY16*, a stress responsive gene induced by drought, salt, alkali, PEG, and ABA, was analyzed for its functional characterization of stress tolerance in Arabidopsis. Transgenic Arabidopsis plants of *GmWRKY16* enhanced drought and salt tolerance as compared to wild type. Furthermore, the regulated genes responding to stresses were investigated in Arabidopsis for potential mechanisms through an ABA-mediated pathway.

## Materials and Methods

### Plant Materials, Growth Conditions, and Treatments

A spring-sowing soybean cultivar, “Huachun 2” (HC2), bred by the Guangdong Subcenter of National Center for Soybean Improvement in South China Agricultural University, China, was used to investigate the tissue expression pattern of *GmWRKY16* and its responses to various stresses including mannitol, salt, drought, and ABA. Soybean seeds of HC2 were germinated, and its seedlings grew in growth chamber under a 12 h light/12 h dark cycle with 100 μM photons m^-2^ s^-1^ at 25–30°C for 3 weeks as described previously by Zeng et al. (2012). The soybean roots of 3-week-old seedlings were immersed in Hoagland solution saturated with 200 mM NaCl, 50 mM NaHCO_3_, 20% (w/v) PEG-6000 or 100 μM ABA, respectively (Luo et al., 2013; Chen et al., 2015). Root samples were harvested after time intervals of 0, 1, 3, 6, 9, 12, and 24 h, respectively. Samples were frozen immediately in liquid nitrogen and subsequently stored for RNA extraction at -80°C.

Ecotype Col-0 of *Arabidopsis* was used as the receptor for genetic transformation of *GmWRKY16* gene. All the Arabidopsis seeds of wild-type and transgenic lines were grown on the agar medium of 1/2 Murashige and Skoog (MS) in darkness for 2 days at 4°C after the seeds were sterilized with ddH_2_o. The seedlings were then cultured for several days under long-day conditions (16 h light/8 h dark cycle) at 22°C in new 1/2 MS medium. The chemical compounds of mannitol, NaCl, and ABA were dissolved in MS medium, respectively. The taken samples of Arabidopsis were frozen immediately in liquid nitrogen and stored at -80°C (Chen et al., 2015).

### *GmWRKY16* Gene Isolation and Sequence Analysis

According to previous reports, the sequence information of the *GmWRKY16* gene involved in abiotic stress was obtained from the database of the National Center for Biotechnology Information (NCBI) with the accession number XP_003518509. The full-length sequence of *GmWRKY16* was amplified by RT-PCR using specific primers (Supplementary Table S1) and Super-Fidelity DNA polymerase (Phanta Max, Vazyme Biotech Co., Ltd.; Nanjing, China). The seedlings of HC2 under the treatment of 100 mM NaCl were used to extract total RNA by TRIzol reagent (Invitrogen). The methods of generated cDNA, RT-PCR reaction and agarose gel electrophoresis were described in detail previously (Lü et al., 2015). The purified PCR product was then inserted into the multiple cloning sites of the pLB vector (Tiangen Rapid DNA Ligation Kit, Beijing, China). The positive clones in *Escherichia coli* were used to obtain the full cDNA sequence of *GmWRKY16* identified by PCR, enzyme digestion and sequencing [Sangon Biotech (Shanghai) Co., Ltd., China] (Lü et al., 2015; Ma et al., 2018).

The nucleotide sequence of *GmWRKY16* and the amino acid sequence of GmWRKY16 protein were used to search its homologous genes and proteins using the database of NCBI^1^. The alignment of nucleotide sequences was performed with the software of DNAMAN. The structure prediction of the GmWRKY16 protein was carried out using the NCBI blast results. Phylogenetic analysis was enforced by using the software of MEGA 7 (Kumar et al., 2016). The alignment was adjusted manually, while the unrooted phylogenetic trees were constructed by the Neighbor-Joining method (Sievers et al., 2011).

### Plasmid Construction and Transformation of *GmWRKY16* in Arabidopsis

The full-length coding sequence of *GmWRKY16* (Supplementary Table S1) amplified from the GmWRKY16-pLB vector was inserted into the *Bam*HI and *Kpn*I sites of a pTF-101 vector using specific primers (Supplementary Table S1). The pTF-101-GmWRKY16 fusion construct with a β-glucuronidase (GUS) reporter was generated under the control of the cauliflower mosaic virus 35S (CaMV 35S) promoter. The pTF-101-GmWRKY16 plasmid was then transformed into *Agrobacterium tumefaciens* GV3101 competent cells by the electroporation method. The full-flowering Arabidopsis plants were used for genetic transformation by the floral dip method (Clough and Bent, 1998). All the *GmWRKY16* transgenic seedlings were screened by an herbicide to obtain the positive plants.

### Subcellular Localization of GmWRKY16 Protein

Localization of the GmWRKY16 protein was carried out using the method described previously (Lü et al., 2015). The complete coding sequence of *GmWRKY16* without a stop codon amplified by PCR using the specific primers was inserted into the *Nco*I and *Spe*I restriction sites to generate a fusion construct under the control of CaMV 35S promoter. The plasmids of pCAMBIA1302 and pCAMBIA1302-GmWRKY16 were transformed into *Agrobacterium tumefaciens* strain GV3101 by the heat shock method. The young leaves of 4-week-old tobacco plants were contaminated by the recombinant *Agrobacterium tumefaciens* using the method according to Kokkirala et al. (2010). The leaves after 48 h agro-infiltration were photographed with a confocal laser scanning microscope (Leica, Germany).

### Transactivation Assay

The full-length cDNA of *GmWRKY16* amplified by PCR using specific primers was inserted into the *Eco*RI and *Sac*I sites to create a fusion construct of pGBKT7-GmWRKY16 (Supplementary Table S1). The pGBKT7-GmWRKY16 plasmid was transformed into yeast strain Y2H. An empty pGBKT7 vector was used as a negative control. Transcriptional activation was analyzed according to the methods described previously (Yang et al., 2010).

### Expression Analysis by qRT-PCR

Total RNA was extracted from the seedlings of soybean or Arabidopsis using the plant total RNA kit (GeneMark, Taiwan, China). Reversing transcription for the first strand cDNA synthesis was performed with 2 μg of total RNA using a Prime Script^TM^ RT reagent Kit (Takara, Beijing, China). The qRT-PCR analyses were performed on a CFX96^TM^ Real-Time system (United States) with SYBR Premix ExTaq II (Takara, Beijing, China). The inner reference gene β-*tubulin* was used to normalize the data. The quantitative variations of gene expression between the examined replicates were evaluated by the 2^-ΔΔCt^ method described previously (Willems et al., 2008; Lü et al., 2015). Three independent biological repeats were performed to ensure accurate statistical analysis. The specific primers were listed in Supplementary Table S1.

### Stress Tolerance Assays of Seed Germination

To detect seed germination of *GmWRKY16* transgenic lines under the condition of mannitol, NaCl or ABA stresses, the surface-sterilized Arabidopsis seeds of wild type and transgenic lines were planted on solid 1/2 MS media with various concentrations of mannitol (0, 100, 200, and 300 mM), NaCl (0, 100, 150, and 200 mM), and ABA (0, 0.5, 1, and 1.5 μM). The rates of seed germination were recorded every day with images taken on the 4th and 7th day after seed was sown. For the root length assay, the sterilized seeds of transgenic lines and WT were grown on 1/2 MS media for 3 days, and then the seedlings were transplanted to fresh media supplemented with mannitol (0, 150, and 300 mM), NaCl (0, 75, and 150 mM), and ABA (0, 0.5, and 1.0 μM) for another 7 days to examine the root length (Rushton et al., 2012; Seo et al., 2012; Shang et al., 2012; Chen et al., 2015). The root elongation of Arabidopsis plants was monitored and analyzed using Image J software^2^.

### Drought and Salt Treatments in Transgenic Arabidopsis

For drought treatments, Arabidopsis seeds of the T_3_ generation of WT and *GmWRKY16* overexpression lines were sown in mixed soil (vermiculite and flower nutrient soil, 1:1) for 3 weeks under optimal irrigation conditions. The following drought stress treatments were set as 5, 7, and 9 days without watering, respectively. All the Arabidopsis plants were watered again for 5 days after their photographs were taken and their survival rates were determined (Seo et al., 2012; Ying et al., 2012; Babitha et al., 2013). For salt treatments, Arabidopsis seeds of the WT and *GmWRKY16* overexpression lines of the T_3_ generation were sown in mixed soil (vermiculite and flower nutrient soil, 1:1) in the chambers. The 3-week-old Arabidopsis plants were then irrigated with a solution of 200 mM NaCl for 15 days (Niu et al., 2012; Ying et al., 2012; Babitha et al., 2013).

### Measurements for Small Molecules and Dehydration

The content of free proline in plants was determined following methods described previously (Bates et al., 1973). Briefly, 0.5 g of plant samples were homogenized in 5 ml of 3% sulfosalicylic acid using a mortar. The 2 ml supernatant was transferred to a new tube, and then 2 ml of acid ninhydrin and 2 ml of glacial acetic acid were added to form the extraction solution. The reaction mixture was boiled in a water bath at 100°C for 1 h and then stored at 4°C for 30 min. After the mixtures were centrifuged at 3,000 *g* for 5 min, the proline content for each sample was measured at 520 nm absorbance. The 3-week-old Arabidopsis seedlings were prepared in accordance with the above description. After treatment of 200 mM NaCl for 1 week, the leaf samples were taken to measure the MDA contents using the method of thiobarbituric acid (TBA) described in detail previously (Heath and Packer, 1968). For measuring dehydration, the leaves of rosettes were cut from the 3-week-old plants and immediately placed on a laboratory bench (40% relative humidity). Water loss of fresh leaves was weighed at treatment intervals set as 0, 0.5, 1, 1.5, 2, 3, 4, 5, and 6 h, respectively. All of the experiments comprised three replicates. ABA content was measured using an enzyme-linked immunosorbent assay (ELISA), as previously described (Yang et al., 2001).

### Data Analysis

All data were presented as the mean of three biological replicates ± SD. Student’s test at *P* = 0.01 or *P* = 0.05 was used to identify the difference between observation values (Lü et al., 2015).

## Results

### Cloning and Bioinformatics Analysis of *GmWRKY16*

The WRKY superfamily is one of the biggest transcription factor (TF) families in soybean and plays multiple roles in plant growth and development, abiotic stresses, etc. (Bencke-Malato et al., 2014; Dias et al., 2016; Yu Y. et al., 2016; Yang et al., 2017). Based on recent reports on the functions of WRKY TFs in soybean (Yu Y. et al., 2016), a salt and/or drought stress-induced gene encoding a transcription factor of WRKY proteins was obtained from the database under gene locus LOC100790175 and protein accession number of XP_003518509. The *WRKY* gene, located on chromosome 2 of soybean and designed as *GmWRKY16*, was cloned using the specific primers (Supplementary Table S1) from the cultivar HC2. The full-length genome sequence of *GmWRKY16* included 3 exons and 2 introns with a full-length cDNA of 1014 bp (data not shown). The predicted GmWRKY16 protein comprises 337 amino acids (AA) with 38.113 kDa of molecular weight (data not shown).

**FIGURE 1:**
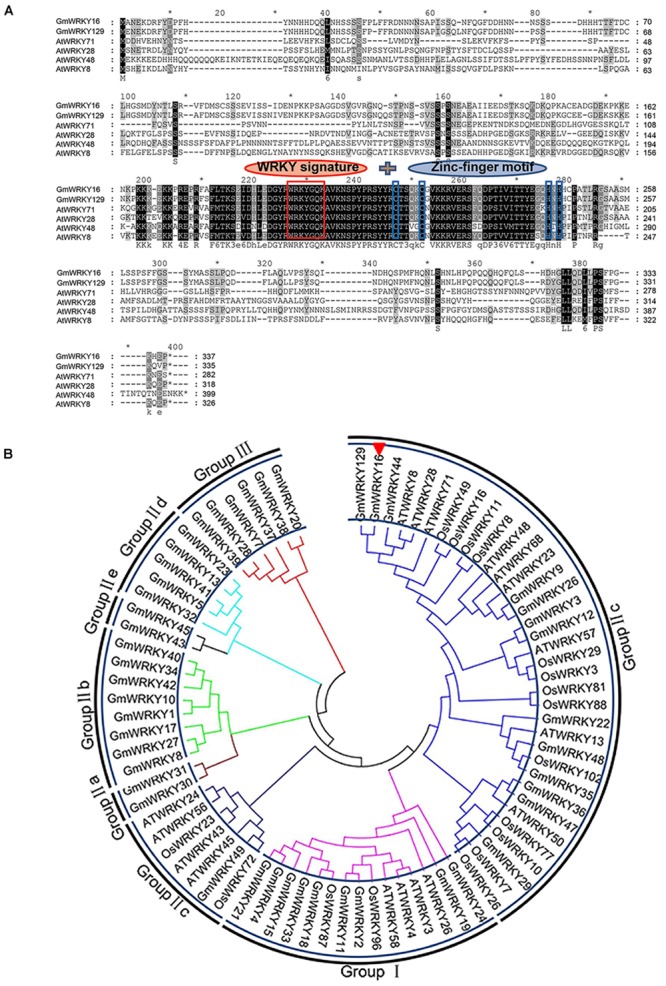
Homology analysis of GmWRKY16 and other WRKY transcription factors. **(A)** Multiple sequence alignment of GmWRKY16 and WRKY family members from Arabidopsis and soybean. **(B)** Phylogenetic tree analysis of GmWRKY16 and WRKY proteins. The comparison of amino acid sequences was conducted by the software of DNAMAN6.0. The phylogenetic tree was constructed by the neighbor-joining method using the software of MEGA 7.0. All the amino acid sequences and the accession numbers of WRKY proteins from soybean, rice and Arabidopsis were obtained from the databases of NCBI (https://www.ncbi.nlm.nih.gov/). The detailed information of WRKY proteins was available from the Supplementary Table S2.

The NCBI BLAST analysis of the full amino acids sequence indicated that the GmWRKY16 protein had a WRKY DNA-binding domain at the location of the peptide chain between 190 AA and 250 AA (data not shown). The results of multiple alignment using the Phytozome data showed that the GmWRKY16 protein contained two small domains with a motif of WRKYGQK and a C_2_H_2_ zinc-finger motif similar to those of five other WRKY proteins from Arabidopsis and soybean (Figure 1A). To investigate the relationship between GmWRKY16 and other WRKY proteins from soybean, rice and Arabidopsis, a phylogenetic tree was constructed by the neighbor-joining method using the MEGA 7.0 software (Figure 1B). The results indicated that all the 80 WRKY proteins were divided into three major groups: Group I (15 proteins), Group II (59 proteins), and Group III (6 proteins). In addition, 59 WRKY proteins in Group II were further divided into five subgroups: Group II-a, Group II-b, Group II-c, Group II-d, and Group II-e. GmWRKY16 protein belongs to the WRKY members of Group II-c with 93% similarity to GmWRKY129 protein (Figure 1B). Therefore, the bioinformatics analysis suggest that GmWRKY16 protein may have a function in abiotic stresses.

### Characteristics of Localization and Transcriptional Activation Ability of GmWRKY16 Protein

**FIGURE 2:**
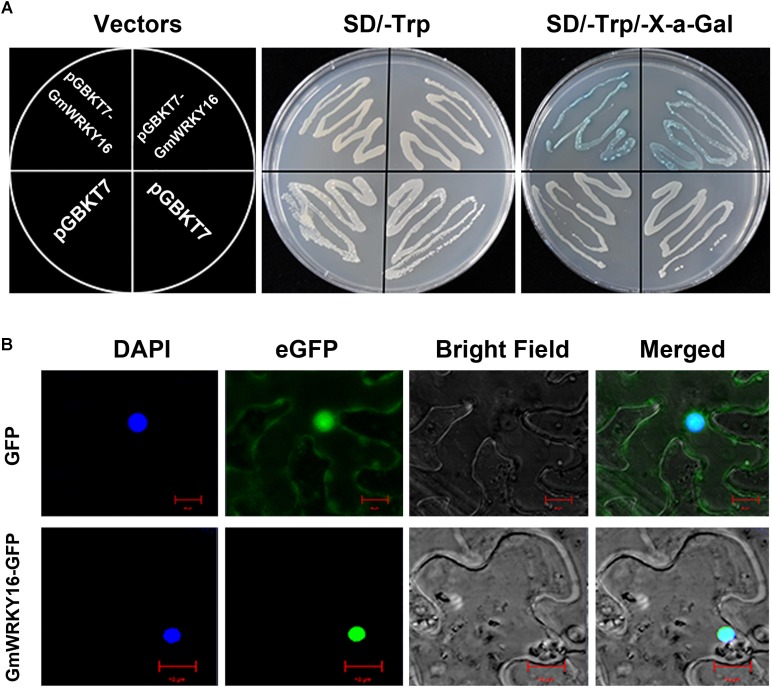
Transcriptional activation and subcellular localization of GmWRKY16 protein. **(A)** Transcriptional activation of GmWRKY16 protein in yeast cells. **(B)** Subcellular localization of GmWRKY16 protein in leaf epidermal cells of tobacco. The ORF sequence of *GmWRKY16* was inserted into the sites of *Eco*RI and *Sac*I of pGBKT7 vector to form the fusion carrier of GmWRKY16-pGBKT7. The competent yeast cells were transformed using the heat-shock method. After being cultured at 30°C for 48 h, the transcriptional activation of GmWRKY16 protein was detected by the method of color reaction in yeast cells using *X*-α-Gal as a substrate. The full coding sequence of GmWRKY16 (without TAA) was inserted into the *Nco*I and *Spe*I sites of pCAMBIA1302 vector to obtain the GmWRKY16-GFP fusion construct. The vectors of pCAMBIA1302 and pCAMBIA1302-GmWRKY16 were transformed into the young leaves of 4-week-old tobacco plants using the method of Kokkirala mediated by *Agrobacterium tumefaciens* (Kokkirala et al., 2010). The protein expression of GFP or GmWRKY16-GFP from the leaves after agro-infiltration for 48 h was visualized using a confocal laser scanning microscope (Leica, Germany) (Zhang et al., 2011).

To determine the transcriptional activity of GmWRKY16 protein, the ORF sequence of *GmWRKY16* was inserted into the *Eco*RI and *Sac*I sites of pGBKT7 to form a GmWRKY16-pGBKT7 construct (data not shown). The fusion plasmid of GmWRKY16-pGBKT7 and pGBKT7 alone (negative control) were transformed into the cells of yeast strain Y2H, respectively. As shown in Figure 2A, yeast cells transformed by the GmWRKY16-pGBKT7 and pGBKT7 vectors could grew well on the SD/-Trp medium without any differences. However, the cell deposits transformed by the GmWRKY16-pGBKT7 construct turned blue on the SD/-Trp/X-α-Gal medium colored with chromogenic substrate of *X*-gal (Figure 2A). These results indicated that GmWRKY16 protein had transcriptional activity in yeast cell.

To verify the subcellular localization of GmWRKY16 protein, the full-length GmWRKY16 cDNA without the termination codon was fused in-frame to the 5′ end of *GFP* gene at the *Nco*I and *Spe*I sites of the pCAMBIA1302 vector to obtain the GmWRKY16-GFP fusion construct (data not shown). The fusion plasmid of pCAMBIA1302-GmWRKY16 and pCAMBIA1302 alone were transformed into the young leaves of 4-week-old tobacco plants, respectively. As shown in Figure 2B, the GmWRKY16-GFP protein accumulated mainly in the nucleus with strong signals of green fluorescence. In contrast, GFP alone was distributed evenly throughout all parts of the cell including the nucleus and cytoplasm (Figure 2B). The result of confocal microscopic analysis suggests that the GmWRKY16 protein may function as a transcription factor.

### Expression Patterns of *GmWRKY16* Under Abiotic Stress Conditions

**FIGURE 3:**
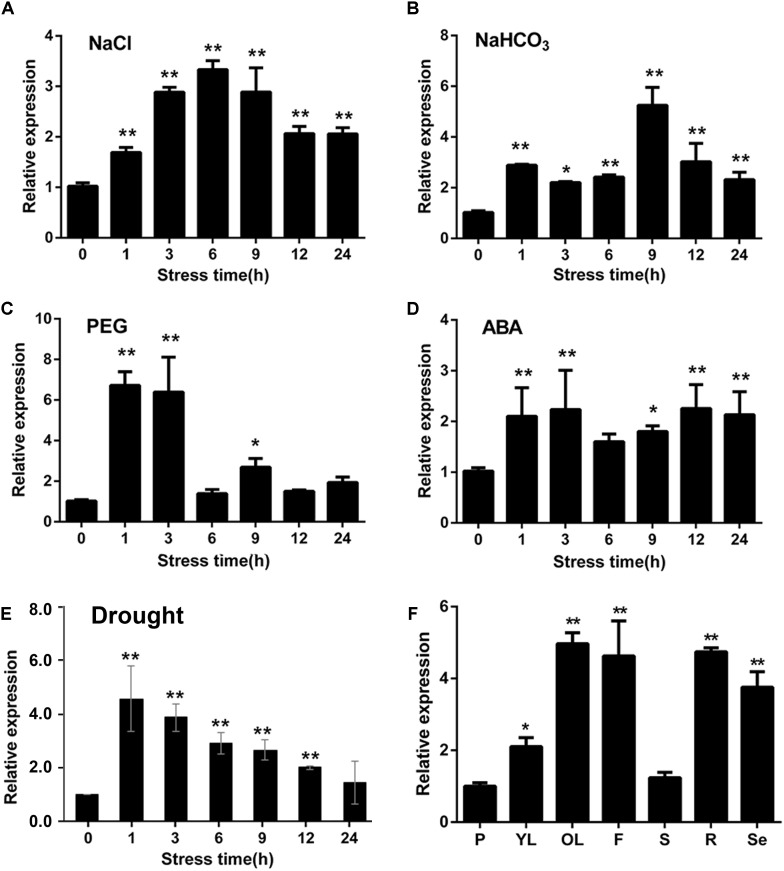
Expression patterns of *GmWRKY16* under different abiotic stress conditions and in soybean tissues. **(A–E)** Patterns of *GmWRKY16* expression under the different conditions of abiotic stresses. **(B)** Tissue expression pattern of *GmWRKY16* in soybean. The soybean roots of 3-week-old seedlings were immersed in Hoagland solution saturated with 200 mM NaCl **(A)**, 50 mM NaHCO_3_ **(B)**, 20% PEG-6000 **(C)**, and 100 μm ABA **(D)**, respectively. Root samples were harvested at 0, 1, 3, 6, 9, 12, and 24 h after different treatments, respectively. The 1-week-old seedlings were dehydrated at room temperature **(E)**, while the seedling samples were taken at treatment times of 0, 1, 3, 6, 9, 12, and 24 h, respectively. **(F)** Tissue samples were taken at different stages of soybean growth and development. *GmWRKY16* transcript abundance was assessed by qRT-PCR using the 2^-ΔΔCt^ method with the actin *ACT3* gene as an internal control (Lü et al., 2015). The data are represented as the averages of three independent biological experiments ± SD, and asterisks indicate a significant difference (^∗^*P* = 0.05; ^∗∗^*P* = 0.01) compared with the corresponding controls. P, young pod; YL, young leaf; OL, old leaf; F, flower; S, stem; R, root; Se, seed.

To investigate the expression patterns of *GmWRKY16* response to abiotic stresses, quantitative real-time PCR (qRT-PCR) was performed to analyze the transcript abundance of *GmWRKY16* under salt, alkali, PEG-6000, ABA and drought treatments. As shown in Figure 3A, *GmWRKY16* was quickly induced by 200 mM NaCl with increasing expression levels. Total RNA of *GmWRKY16* was increased up to the highest level of 3.5-fold at the 6 h treatment in roots compared to that of the control, and then declined to a lower level under the 9 h to 24 h treatments. Similarly, under treatment of 50 mM NaHCO_3_, *GmWRKY16* increased slowly under the treatments from 1 to 9 h up to the highest expression level of 5.8-fold (Figure 3B). Meanwhile, *GmWRKY16* was induced rapidly by PEG-6000 up to the highest expression level of 6.8-fold (Figure 3C). For ABA treatment, *GmWRKY16* was also induced rapidly up to its highest expression level of 2.5-fold, and then the expression of *GmWRKY16* maintained at a relatively high level (Figure 3D). Furthermore, the expression of *GmWRKY16* was increased along with the treatments of dehydration with its highest expression level of 4.6-fold (Figure 3E). These findings suggest that *GmWRKY16* might play a role in multiple abiotic stresses.

To detect the tissue expression pattern of *GmWRKY16*, the leaf, stem, pod, flower, root, and seed samples were taken from the soybean cultivar HC2. The analysis of qRT-PCR indicated that *GmWRKY16* was expressed constitutively in soybean with a more than fivefold expression level in old leaves compared to those in the stem and young pod (Figure 3F). In addition, *GmWRKY16* has higher expression of more than 3.8-fold levels in soybean organs including flower, root, and seed (Figure 3F). These results suggest that *GmWRKY16* may have some functions in soybean tissues and/or organs.

### *GmWRKY16* Improved the Tolerance of Transgenic Arabidopsis to Osmotic Stress

**FIGURE 4:**
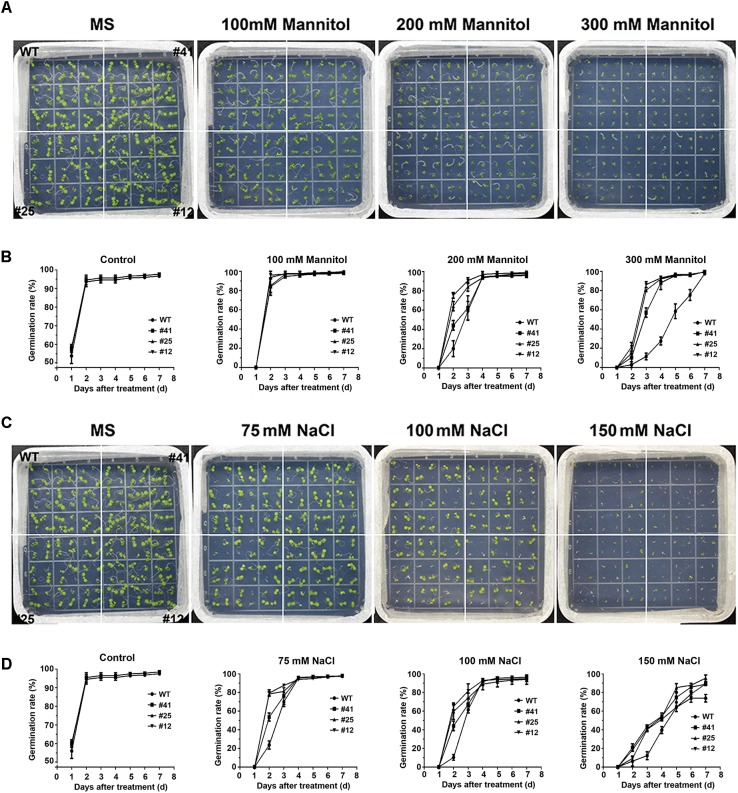
Overexpression of *GmWRKY16* increased the germination rates of Arabidopsis under the conditions of osmotic stress. **(A,B)** Seed germination under the treatment of mannitol. **(C,D)** Seed germination under the treatment of salt. The surface-sterilized Arabidopsis seeds of wild-type and transgenic lines were sown on solid media of 1/2 MS containing mannitol (0, 100, 200, and 300 mM) and NaCl (0, 100, 150, and 200 mM). The status of seed germination was taken by photos after the treatment of osmotic stress for 4 and 7 days, respectively, while the germination rate of seeds was counted every day. Error bars represent ± SD. The observation values were the averages of three repetitions (*n* = 3). Three independent biological experiments were carried out to investigate the seed germination of WT and *GmWRKY16* transgenic lines under osmotic stress. WT, wild type; #12, 25, 41: *GmWRKY16* Arabidopsis transgenic lines of T_3_ generations.

**FIGURE 5:**
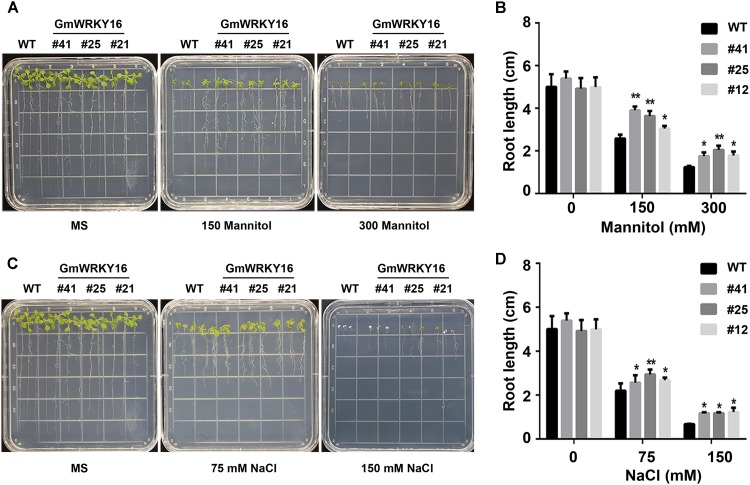
Overexpression of *GmWRKY16* improved the tolerance of Arabidopsis seedlings to osmotic stress. **(A,B)** Seedlings under the treatment of mannitol. **(C,D)** Seedlings under the treatment of salt. The surface-sterilized Arabidopsis seeds of wild type and transgenic lines were sown on the solid media of 1/2 MS without mannitol and NaCl. The 3-day-old seedlings from 1/2 MS medium were transferred to the plates of 1/2 MS containing mannitol (0, 150, and 300 mM) and NaCl (0, 75, and 200 mM). The status of seedlings was taken by photos and root lengths were measured when Arabidopsis plants were treated under osmotic stress for 7 days. The vertical columns for the average observation value of the three repetitions represent the means ± SD. Three independent biological experiments were carried out to investigate the status of seedlings and root growth of WT and *GmWRKY16* transgenic lines under osmotic stress. Asterisks indicate significant differences between WT and *GmWRKY16* transgenic lines (^∗^*P* = 0.05; ^∗∗^*P* = 0.01). WT, wild type; #12, 25, 41: *GmWRKY16* Arabidopsis transgenic lines of T_3_ generations.

To investigate the tolerance to osmotic stress of *GmWRKY16* transgenic lines after molecular identification (Supplementary Figure S1), different treatments of mannitol and NaCl were performed on 1/2 MS medium to determine the germination rates and root elongation of Arabidopsis (Figures 4, 5). Statistical results showed that seed germination of Arabidopsis was inhibited by mannitol stress (Figures 4A,B). As compared with the control (0 mM mannitol), the transgenic Arabidopsis lines of *GmWRKY16* overexpression enhanced seed germination rates at 100, 200, and 300 mM of mannitol, respectively. The germination rates of *GmWRKY16* transgenic lines were almost up to 100% under the mannitol treatments at the 3rd, 4th, and 5th days, respectively. The wild-type germination was severely suppressed, with lower germination rates compared to those of *GmWRKY16* transgenic lines under treatments of 200 mM and 300 mM mannitol, respectively (Figures 4A,B). When Arabidopsis seeds were treated with NaCl, *GmWRKY16* transgenic plants germinated faster than those of WT in the first 3 days. However, no significant differences in germination rate were found between WT and *GmWRKY16* transgenic lines after 3 days under the treatments of 75, 100, and 150 mM NaCl, respectively (Figures 4C,D).

The root lengths of Arabidopsis were inhibited by mannitol and NaCl stresses with the increase of the treatment concentrations (Figure 5). The root lengths of WT plants were 2.5 cm long under treatment of 150 mM mannitol, which was almost half the length of the control, while the root lengths of *GmWRKY16* transgenic lines were more than 3 cm up to 4.1 cm long under treatment of 150 mM mannitol (Figures 5A,B). Under treatment of 300 mM mannitol, the root lengths of WT and of *GmWRKY16* transgenic lines were inhibited to 1.5 cm and more than 2 cm long, respectively (Figures 5A,B). In the meantime, the inhibition of root elongation was also found under salt stress (Figures 5C,D). Compared to the control, the root lengths of WT were 2.2 and 0.7 cm long under the treatments of 75 and 150 mM NaCl, respectively, while the root lengths of *GmWRKY16* transgenic lines were over 2.5 and 1.1 cm long under the corresponding treatments (Figures 5C,D). These results suggest that overexpression of *GmWRKY16* enhances tolerance to osmotic stress.

**FIGURE 6:**
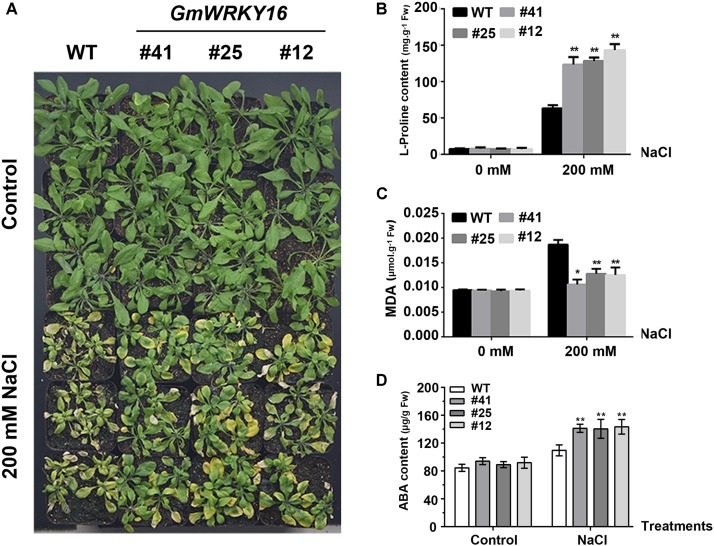
Overexpression of *GmWRKY16* in Arabidopsis enhanced salt tolerance. **(A)** The phenotypes of *GmWRKY16* transgenic lines tolerant to salt stress. **(B)** The determination of free proline content. **(C)** The determination of MDA content. **(D)** The determination of ABA content. Arabidopsis seeds of WT and *GmWRKY16* transgenic lines of T_3_ generation were sown in mixed soil (vermiculite and flower nutrient soil, 1:1) and cultured in the chamber room. The 3-week-old Arabidopsis plants were then irrigated with a solution of 200 mM NaCl for 15 days to determinate the contents of free proline, MDA and ABA. The vertical columns for the average observation value of the three repetitions represent the means ± SD. Three independent biological experiments were carried out to investigate the status of seedlings and accumulations of proline, MDA and ABA in plants of WT and *GmWRKY16* transgenic lines under salt stress. Asterisks indicate significant differences between WT and *GmWRKY16* transgenic lines (^∗^*P* = 0.05; ^∗∗^*P* = 0.01). WT, wild type; #12, 25, 41: *GmWRKY16* Arabidopsis transgenic lines of T_3_ generations.

To test the performance of the adult transgenic plants under NaCl stress, 21-day-old Arabidopsis plants were irrigated with a solution of 200 mM NaCl for 15 days. We observed that the leaves of the wild type were severely affected by salinity, whereas the leaves of *GmWRKY16* transgenic lines were less affected (Figure 6A). It was found that 200 mM NaCl treatment induced proline accumulation both in wild-type plants and *GmWRKY16* transgenic lines, with 65 and over 135 mg.g^-1^ of fresh weight, respectively (Figure 6B). In contrast, a greater MDA accumulation of 0.019 μmol.g^-1^ of fresh weight was found in WT compared to those of *GmWRKY16* transgenic lines, with less than 0.013 μmol.g^-1^ of fresh weight under salt stress (Figure 6C). Meanwhile, ABA accumulation of over 130 μg.g^-1^ of fresh weight was determined in *GmWRKY16* transgenic plants compared to those of WT, with less than 100 μg.g^-1^ of fresh weight (Figure 6D).

### *GmWRKY16* Enhanced the Tolerance of Transgenic Arabidopsis to Drought Stress

**FIGURE 7:**
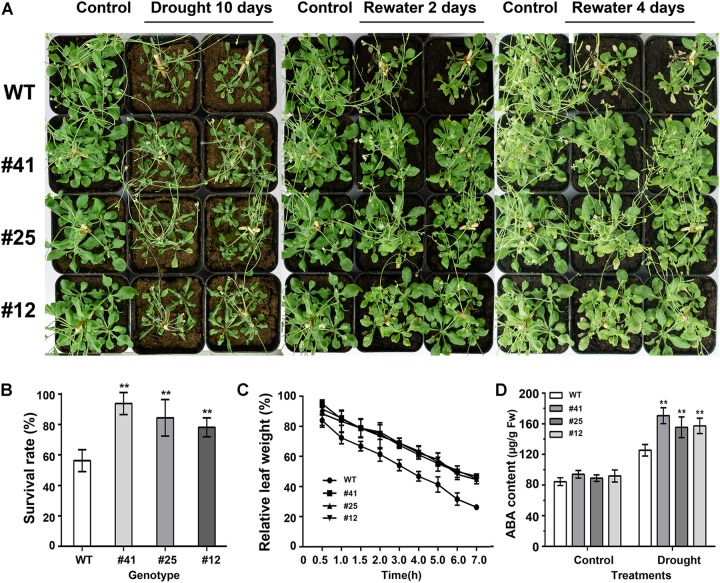
Overexpression of *GmWRKY16* Arabidopsis conferred drought tolerance. **(A)** The phenotypes of *GmWRKY16* transgenic lines tolerant to drought stress. **(B)** The survival rates of Arabidopsis plants. **(C)** The water loss of detached leaves. **(D)** The determination of ABA content. Arabidopsis seeds of WT and *GmWRKY16* transgenic lines of T_3_ generation were sown in mixed soil (vermiculite and flower nutrient soil, 1:1) and cultured in the chamber room. The 3-week-old Arabidopsis plants were subjected to water shortage for 5, 7, and 9 days, respectively. All the drought-treated plants were then rehydrated after they were dried for 5 days. The fresh weight was measured at the set intervals of drought stress. The vertical columns for the average observation value of the three repetitions represent the means ± SD. Three independent biological experiments were carried out to investigate the status of seedlings, the survival rate and water loss of the detached leaves of WT and *GmWRKY16* transgenic lines under drought stress. Asterisks indicate the significant differences between WT and *GmWRKY16* transgenic lines (^∗^*P* = 0.05; ^∗∗^*P* = 0.01). WT: wild type; #12, 25, 41: *GmWRKY16* Arabidopsis transgenic lines of T_3_ generations.

To test plant response to drought stress, 21-day-old Arabidopsis plants under optimal irrigation conditions were treated without watering for 6, 8, or 10 days, respectively. The observation results showed that Arabidopsis plants were damaged by water shortage. As shown in Figure 7A, WT plants were severely damaged, with symptoms of leaf yellowing and crimping as well as a 60% survival rate after 10 days of drought treatment (Figure 7B), while *GmWRKY16* transgenic plants grew well with an over 80% survival rate (Figure 7B). Furthermore, detached leaves of WT lost water much faster than those of *GmWRKY16* transgenic lines under the dehydration treatment (Figure 7C). Meanwhile, ABA accumulation of over 150 μg.g^-1^ of fresh weight was determined in *GmWRKY16* transgenic plants compared to those of WT, with less than 120 μg.g^-1^ of fresh weight (Figure 7D). Therefore, the data indicated that overexpression of *GmWRKY16* enhanced Arabidopsis tolerance to drought stress.

### *GmWRKY16* Conferred the Tolerance of Transgenic Arabidopsis to ABA

**FIGURE 8:**
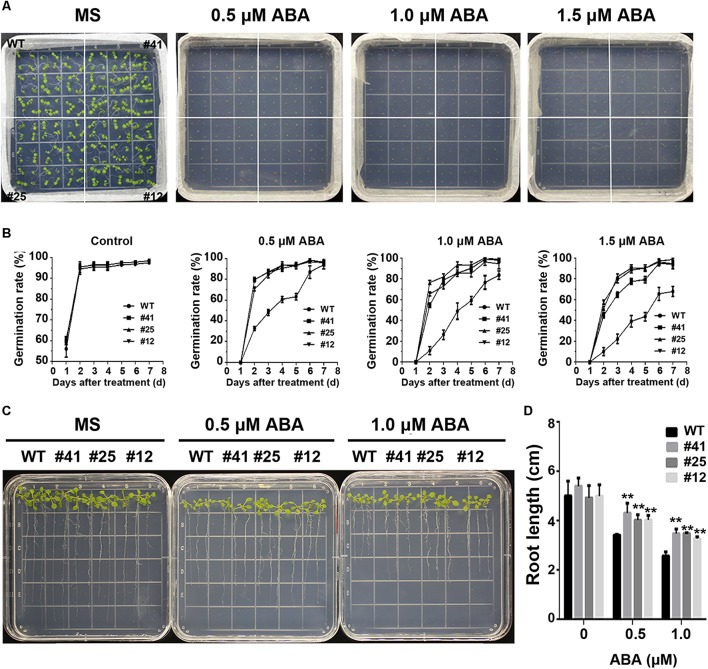
Overexpression of *GmWRKY16* in Arabidopsis confers the tolerance to ABA. **(A,B)** Seed germination under ABA treatment. **(C,D)** Seedlings under ABA treatment. The surface-sterilized Arabidopsis seeds of wild-type and transgenic lines were sown on the solid media of 1/2 MS containing ABA (0, 0.5, 1.0, and 1.5 μM). The photos were taken for the status of seed germination after the ABA treatment for 4 and 7 days, respectively. The 3-day-old seedlings from 1/2 MS medium were transferred to the plates of 1/2 MS containing ABA (0, 0.5, and 1.0 μM). The root lengths were measured when Arabidopsis plants were treated under the ABA stress for 7 days. Error bars represent ± SD. The observation values were the averages of three repetitions. Three independent biological experiments were carried out to investigate the seed germination and root growth of WT and *GmWRKY16* transgenic lines under ABA stress. WT, wild type; #12, 25, 41: *GmWRKY16* Arabidopsis transgenic lines of T_3_ generations.

To study *GmWRKY16* sensitivity to ABA, different treatments of ABA were performed on 1/2 MS medium to determine the germination rates and root elongation of Arabidopsis (Figure 8). The statistical results showed that seed germination of Arabidopsis was inhibited by ABA stress with increasing concentrations (Figures 8A,B). When compared to the control (0 μM ABA), the *GmWRKY16* transgenic lines enhanced seed germination rates under ABA treatments of 0.5, 1.0, and 1.5 mM, respectively. The germination rates of *GmWRKY16* transgenic lines were almost up to 100% under the ABA treatments at the 6th day. Meanwhile, the germination rates of the wild type were less than 90, 80, and 70% with increasing concentrations under ABA treatments (Figures 8A,B).

The root lengths of Arabidopsis were also inhibited by ABA stress (Figures 8C,D). The root lengths of WT plants were 3.5 cm long under the treatment of 0.5 μM ABA, or around 1.8 cm shorter than that under the control, while the root lengths of *GmWRKY16* transgenic lines were over 4.1 cm long under the treatment of 0.5 μM ABA (Figures 8C,D). Under the treatment of 1 μM ABA, the root elongations of WT and *GmWRKY16* transgenic lines were also inhibited with 2.8 cm and over 3.2 cm long, respectively (Figures 8C,D). These results suggest that overexpression of *GmWRKY16* enhances the Arabidopsis tolerance to ABA stress.

### Expression Patterns of Stress/ABA Responsive Genes Regulated by *GmWRKY16*

**FIGURE 9:**
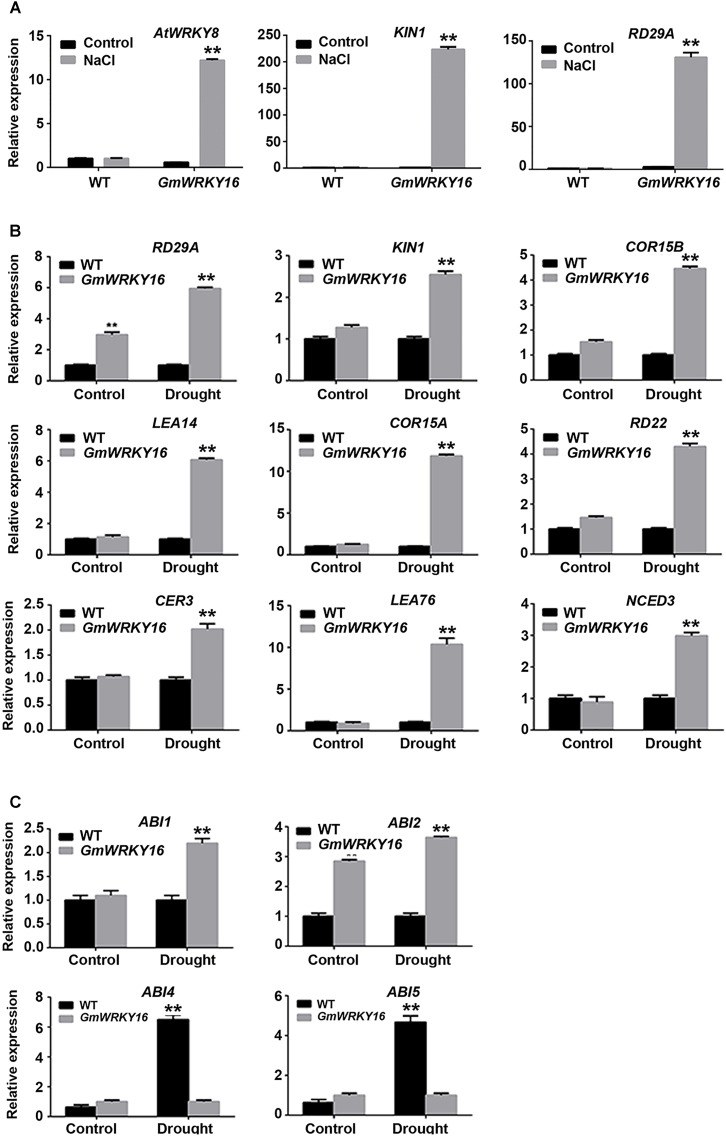
Expression patterns of stress/ABA responsive genes regulated by *GmWRKY16*. **(A)** Expression patterns of genes responsive to salt stress. **(B)** Expression patterns of ABA and/or stress-responsive genes under drought stress. **(C)** Expression patterns of ABA-responsive genes under drought stress. Arabidopsis seeds of WT and *GmWRKY16* transgenic line #12 of T_3_ generation were sown in mixed soil (vermiculite and flower nutrient soil, 1:1) and cultured in the chamber room. The 2-week-old seedlings were subjected to drought treatment (withholding water) and 200 mM NaCl treatment for 10 days. The samples from the aerial parts of Arabidopsis plants were taken for total RNA extraction. The relative expression of drought- and/or salt stress-responsive genes was quantified by qRT-PCR using *ACT3* as the reference gene to normalize the data (^∗∗^*P* = 0.01). The 2^-ΔΔCt^ method was used to evaluate the quantitative variation between the examined replicates (Lü et al., 2015). The details for the specific primers of the *GmWRKY16*, *ACT3* and stress-responsive genes are listed in Supplementary Table S1.

To further investigate the pathway regulated by *GmWRKY16* under abiotic stress, expression patterns of stress/ABA responsive genes were performed by qRT-PCR. Two genes of *KIN1* and *RD29A* were induced with much higher expression levels, over 220- and 130-fold greater under the NaCl treatment compared with those of the control, respectively (Figure 9A). Meanwhile, *AtWRKY8* was induced with an expression level of over 12-fold greater under the NaCl treatment compared to that of the control (Figure 9A).

Under drought stress, the expressions of *COR15A* and *LEA76* were upregulated in *GmWRKY16* transgenic lines that were increased over 10-fold as compared to those in the wild type (Figure 9B). The expressions of *LEA14*, *RD29A*, *KINI*, *COR15B*, *RD22*, *CER3*, and *NCED3* were upregulated in *GmWRKY16* transgenic lines by less than sixfold as compared to those of wild type (Figure 9B). Among all the detected genes, *RD29A* was induced by *GmWRKY16* in transgenic lines with about a sixfold increased expression level in comparison to that of the wild type (Figure 9B). *ABI2* was induced by *GmWRKY16* in transgenic lines, with 3.6- and 2.8-fold increased expression levels under the drought treatment and the control, respectively. However, *ABI1* was induced by *GmWRKY16* in transgenic lines with a 2.2-fold increased expression level under the drought treatment (Figure 9C). In contrast, two drought-induced genes of *ABI4* and *ABI5* were inhibited with much lower expression levels under the treatment of drought stress (Figure 9C). All the results suggest that *GmWRKY16* may enhance Arabidopsis resistance to salt and drought stresses through ABA and/or other pathways.

## Discussion

The WRKY TFs constitute one of the biggest transcription factor families, which play multiple roles in plants. Since the *SPF1* gene was firstly discovered in sweet potato (Ishiguro and Nakamura, 1994), some WRKY family genes have been investigated for their functional roles and mechanisms involved particularly in biotic and abiotic stresses (Dai et al., 2016; Kiranmai et al., 2018). However, little is known about the WRKYs functional roles and their mechanisms in soybean. In this study, *GmWRKY16* encoding a TF protein of the soybean WRKY family was cloned to determine its potential role in soybean resistance to salt and drought stresses (Figures 6, 7). The phylogenetic tree analysis of GmWRKY16 and other WRKY proteins from soybean, rice and Arabidopsis showed that GmWRKY16 protein in subgroup IIc had more similarity to GmWRKY129 and other WRKY proteins such as AtWRKY8, AtWRKY28, AtWRKY48, AtWRKY71 (Figure 1B). Previous reports indicated that AtWRKY8 enhanced the tolerance of Arabidopsis to salinity stress through modulating its interacting partner VQ9 protein (Hu et al., 2013), that AtWRKY28 conferred resistance to abiotic stress in Arabidopsis by co-expression of *AtbHLH17* (Babitha et al., 2013), and that AtWRKY71 hastened flowering in *Arabidopsis thaliana* in the presence of salt stress (Yu et al., 2018). Furthermore, the multiple alignment of amino acid sequence indicated that GmWRKY16 protein had a conserved WRKY motif and a zinc-finger motif at the central region with characteristics similar to other five WRKY proteins (Figure 1A). The analysis of the biochemistry characteristics of GmWRKY16 protein indicated that GmWRKY16 localizing to the nucleus could activate the expression of GFP in leaf epidermal cells of tobacco and held the transcriptional activation in yeast cells (Figure 2). Therefore, the results above suggest that GmWRKY16 protein may regulate various functions as a WRKY transcription factor involved in abiotic stress and/or development in plants.

Previous studies revealed that WRKY TFs were widely investigated for key roles in abiotic stress responses to drought and salt stresses (Zhou et al., 2008; Wang et al., 2015; Liu et al., 2016). In the present study, the WRKY gene *GmWRKY16* in soybean was quickly induced to the highest expression levels at 3–6 h after treatments of NaCl, PEG, drought, and ABA, respectively (Figure 3). Overexpression of *GmWRKY16* in Arabidopsis promoted seed germination and root elongation of seedlings under NaCl and mannitol treatments (Figures 4, 5), and enhanced the tolerance of transgenic plants to salt and drought stresses (Figures 6, 7). Similar resistant phenotypes to abiotic stress were also found in other WRKY TF genes in soybean. For example, *GmWRKY54* enhanced the tolerance of transgenic Arabidopsis plants to salt and drought stresses through the regulation of *DREB2A* and *STZ/Zat10* (Zhou et al., 2008). Furthermore, *GmWRKY27* was found to improve tolerance to salt and drought stress in soybean plants by interacting with *GmMYB174* (Wang et al., 2015). Recent research indicates that abiotic stresses of drought and salt impose osmotic stresses, which result in the enhanced production of ROS that cause lipid peroxidation, protein oxidation, nucleic acid damage, activation of programmed cell death and then cell death. Meanwhile, it was found that transgenic plants enhanced tolerance to drought as well as osmotic and salt stress through the scavenging of ROS (Foyer and Noctor, 2005; Sharma et al., 2012). The MDA level was an indicator of ROS destructive effects under stress conditions (Sharma et al., 2012), and the content of H_2_O_2_ and MDA in overexpressing transgenic plants were significantly reduced under salt and osmotic stresses (Sairam et al., 1998; Ullah et al., 2018). Proline as an osmolyte plays important roles in stabilizing macromolecules and membranes in cells by a higher accumulation in transgenetic plants under salt and osmotic stress (Wang et al., 2003; Mahajan and Tuteja, 2005; Couée et al., 2006; Cao et al., 2009; Ullah et al., 2018). The contents of proline and MDA in *GmWRKY16* transgenic lines and the wild type were also determined to detect the response of Arabidopsis to drought and salt stresses (Figures 6, 7), and the results were consistent with those of previous reports (Ding et al., 2014; Wang et al., 2015; Yu L. et al., 2016; Ullah et al., 2018), suggesting that overexpression of *GmWRKY16* may enhance the tolerance of drought and salinity by the accumulation of proline and the reduction of MDA in transgenic plants.

Recently, mores studies have indicated that WRKY TFs play vital roles in various responses to abiotic stresses in plants with the potential tolerance mechanisms related to ABA signaling (Zhou et al., 2008; Jiang et al., 2012; Luo et al., 2013; Sun et al., 2015; Cai et al., 2017). Some physiological processes such as seed germination, seedling growth and plant development under various stresses may be inhibited by the accumulation of ABA induced through the ABA-dependent signaling pathway (Xiong et al., 2006; Wang et al., 2014; Wu M. et al., 2017). Overexpression of *GmWRKY16* resulted in better seed germination and longer roots of seedlings under the stress treatments of NaCl, mannitol and exogenous ABA compared to those of the wild type (Figures 4, 5, 8). Moreover, the transgenic Arabidopsis plants exhibited a distinct resistance to salt and drought stresses with higher accumulations of ABA in comparison with the WT plants (Figures 6, 7). Furthermore, overexpression of *GmWRKY16* caused a reduction of stomatal aperture under ABA treatments (Supplementary Figure S2) and decreased water loss rate under drought treatment (Figure 7). There are some reports about ABA role in tolerance to stress. For example, it was found that stoma controlling transpiration water loss in plants is an important factor related to plant resistance to drought stress through changes in ABA level (Holbrook et al., 2002); Luo et al. (2013) found that overexpression of *GsWRKY20* exhibited great tolerance of transgenic Arabidopsis plants to drought stress by decreasing stomatal density under ABA treatment (Luo et al., 2013); and the cotton WRKY transcription factor GhWRKY17 regulated the increased sensitivity of transgenic nicotiana plants to drought stress by reducing the ABA level (Yan et al., 2014). All these results suggest that *GmWRKY16* may enhance plant tolerance to salt, osmotic and drought stresses through positive regulation of ABA pathways. Similar observations were found in other plant species (Zhou et al., 2008; Wu M. et al., 2017; Ullah et al., 2018; Wang et al., 2018).

Some studies have demonstrated that several WRKY TF genes enhanced tolerance to salt and drought stresses through expression regulation of stress/ABA-responsive genes (Yoshida et al., 2014; He et al., 2016; Jaffar et al., 2016; Ullah et al., 2018). To gain insight into the molecular function basis of *GsWRKY16* in salt and drought stress responses, we detected several well-characterized marker genes in transgenic lines (Figure 9). The qRT-PCR results of gene expression showed that *AtWRKY8* and two stress-related marker genes of *KIN1* and *RD29A* were significantly upregulated in transgenic Arabidopsis plants under salt treatment (Figure 9A). Previous reports indicated that AtWRKY8 directly bound the promoter of RD29A to modulate salinity stress tolerance (Hu et al., 2013), suggesting that *GmWRKY16* may function in an ABA-independent pathway. NCED3 (9-*cis* epoxycarotenoid dioxygenase 3) encoding a key and rate-limiting enzyme was supposed to participate in ABA biosynthesis to regulate the drought-stress response (Qin and Jan, 1999; Thompson et al., 2000; Seki et al., 2003). Under drought conditions, NCED3 was determined in *GmWRKY16* transgenic plants with higher levels than those in the WT plants as a result of ABA accumulation (Figures 7D, 9B). Similar results were also found in other WRKY genes, such as *AtWRKY57*, which conferred drought tolerance of Arabidopsis by elevating ABA levels induced by NCED3 (Jiang et al., 2012). These results suggest that *GmWRKY16* may play a role in stress-induced ABA-biosynthesis along with additional factors. The *ABI1* and *ABI2* genes encode two serine/threonine phosphatases 2C (PP2C) proteins that acted as negative regulators of ABA response (Merlot et al., 2001), and the *ABI4* and *ABI5* genes are the best characterized positive regulators of ABA signaling (Reeves et al., 2011; Rushton et al., 2012). In this study, *ABI1* and *ABI2* were upregulated by *GmWRKY16*, whereas *ABI4* and *ABI5* were downregulated in transgenic lines under drought stress (Figure 9C). Previous reports indicated that there was no positive correlation between the gene expression of the ABA signaling pathway and the stress tolerance of transgenic plants to abiotic stresses. Several reports showed that some transgenic plants increased stress tolerance with downregulation of ABI1/2, while other transgenic plants decreased stress tolerance with upregulation of ABI1/2 (Jung et al., 2007; Seo et al., 2010). However, contrary phenotypes of drought tolerance by regulating ABI1/2 have also been reported over the past several years. For example, overexpression of *GmbZIP44*, *GmbZIP62*, or *GmbZIP78* enhanced more tolerance of transgenic Arabidopsis plants to abiotic stress, which may play an important role in ABA signaling by upregulation of ABI1 and ABI2 (Liao et al., 2008). Overexpression of *GsWRKY20* in Arabidopsis increased the tolerance of transgenic plants to drought stress by regulating the expression of some key ABA signaling regulators with upregulation of *ABI*1/2 and downregulation of ABI4/5 (Luo et al., 2013). However, *ZmPP2C* transgenic plants decrease tolerance to salt and drought stresses with lower expression of *ABI*1 and *ABI*2 (Liu et al., 2009). Furthermore, ABREs were detected in a number of ABA-responsive genes (*RD29*, *COR15*, and *RD22*) in several plant species (Kim, 2005). A group of LEA genes, which play important roles in dehydration tolerance (Ingram and Bartels, 1996), was also investigated. In the present study, overexpression of *GmWRKY16* promoted drought tolerance of transgenic plants through regulating expression levels of these stress-related marker genes including *RD29A*, *KINI*, *COR15A*, *COR15B*, *RD22*, *CER3, LEA14*, and LEA76 (Figure 9B). All of these results suggest that overexpression of *GmWRKY16* in Arabidopsis improves the tolerance of transgenic plants to drought and salt stresses through inducing ABA synthesis and regulating the expression levels of ABA signaling and/or stress-related genes.

## Conclusion

In summary, we identified a soybean WRKY transcription factor, GmWRKY16, which is a multiple stress-inducible gene induced by salt drought and ABA stresses and enriched in the old leaves, flowers, seeds, and roots of soybean. *GmWRKY16* overexpression enhanced the resistance of transgenic plants to drought and salt stresses, and less sensitivity to ABA during the processes of seed germination and seedling root growth. The analysis of molecular mechanisms revealed that enhanced tolerance to drought and salt might result in comprehensive roles in upregulating and/or downregulating transcripts of the stress- and/or ABA-responsive genes with ABA and proline accumulation, and MDA decrease. Therefore, these results suggest that GmWRKY16 may increase the resistance to drought and salt stresses through ABA-mediated and/or independent pathways.

## Author Contributions

QM and HN conceived of and designed the study. ZX, QM, ZC, LL, and JL conducted the experiments. ZX, QM, and YC performed data as well as statistical analysis. QM and ZX prepared the manuscript. All authors read and approved the final manuscript.

## Conflict of Interest Statement

The authors declare that the research was conducted in the absence of any commercial or financial relationships that could be construed as a potential conflict of interest.

## Footnotes

1 https://blast.ncbi.nlm.nih.gov/Blast.cgi

2 http://rsb.info.nih.gov/ij
