# Supplementary material for: GmWRKY16 Enhances Drought and Salt Tolerance Through an ABA-Mediated Pathway in Arabidopsis thaliana
Source: Front Plant Sci. 2019 Jan 21;9:1979. doi: 10.3389/fpls.2018.01979 (PMC6357947; doi:10.3389/fpls.2018.01979)
Supplement: Supplementary file 1 [file Table_1.DOCX]

**Table S1.The primers used in the experiment**

| **Primer names** | **Primer sequences (5'to3')** |
| --- | --- |
| Actin-F | TTACCCGATGGGCAAGTC |
| Actin-R | GCTCATACGGTCAGCGATAC |
| ABI1-F | AGAGTGTGCCTTTGTATGGTTTTA |
| ABI1-R | CATCCTCTCTCTACAATAGTTCGCT |
| ABI2-F | GATGGAAGATTCTGTCTCAACGATT |
| ABI2-R | GTTTCTCCTTCACTATCTCCTCCG |
| ABI4-F | ACTCCAAGTTCCGTTACCGTG |
| ABI4-R | GGGGTTAAGTTGAGCTGAGCA |
| ABI5-F | CAATAAGAGAGGGATAGCGAACGAG |
| ABI5-R | CGTCCATTGCTGTCTCCTCCA |
| CER3-F | CTATTGCTCTTTACCTTTGTCGC |
| CER3-R | GAGCAGCATTGTATTTGGTCACT |
| RD29A-F | GGCGTAACAGGTAAACCTAGAG |
| RD29A-R | TCCGATGTAAACGTCGTCC |
| RD22-F | GGTTCGGAAGAAGCGGAG |
| RD22-R | GAAACAGCCCTGACGTGATAT |
| NCED3-F | CAGCTTGTAGCTTTTGGGCTGTA |
| NCED3-R | TAACAGAAACCAGCTGAGCTCGA |
| COR15A-F | GGCCACAAAGAAAGCTTCAG |
| COR15A-R | CTTGTTTGCGGCTTCTTTTC |
| KIN1-F | AACAAGAATGCCTTCCAAGC |
| KIN1-R | CGCATCCGATACACTCTTTCC |
| LEA14-F | GTCATTCGATTCCGATCTGTGAGATC |
| LEA14-R | CCCTACAACAGGAAGGTCGATGG |
| COR15B-F | TCAGTGGCATGGGTTCTTCTTCCA |
| COR15B-R | GAGGTCATCGAGGATGTTGCCGT |
| LEA76-F | GGTGAAGCACACTTTAGGGC |
| LEA76-R | TTCCTCTGTGTCTCACGAGTAGT |
| ACT3-F | GTGCACAATTGAACCAG |
| ACT3-R | GCACCACCGGAGAGAAAATA |
| Q-WRKY-F | CCTCCATTAATGAAAACCCC |
| Q-WRKY-R | TCTCCATCAGCTTCACACGC |
