# Supplementary material for: GmWRKY16 Enhances Drought and Salt Tolerance Through an ABA-Mediated Pathway in Arabidopsis thaliana
Source: Front Plant Sci. 2019 Jan 21;9:1979. doi: 10.3389/fpls.2018.01979 (PMC6357947; doi:10.3389/fpls.2018.01979)
Supplement: Supplementary file 2 [file Table_2.DOCX]

**Table S2. The information of *WRKY* genes from rice, Arabidopsis and soybean**

| **Serial number** | **Gene** | **Gene ID** | **Locus tag** | **Descriptions** | **References** |
| --- | --- | --- | --- | --- | --- |
| 1 | *ATWRKY71* | 839864 | *AT1G29860* | Response to salt tolerant | Yu Y, Wang L, Chen J, et al. WRKY71 Acts Antagonistically Against Salt-Delayed Flowering in Arabidopsis thaliana. Plant and Cell Physiology, 2018, 59(2): 414-422. |
| 2 | *ATWRKY28* | 827542 | *AT4G18170* | Response to drought tolerant | Babitha K C, Ramu S V, Pruthvi V, et al. Co-expression of AtbHLH17 and AtWRKY28 confers resistance to abiotic stress in Arabidopsis. Transgenic research, 2013, 22(2): 327-341. |
| 3 | *ATWRKY48* | 835012 | *AT5G49520* | response to bacterium and chitin | Xing D H, Lai Z B, Zheng Z Y, et al. Stress-and pathogen-induced Arabidopsis WRKY48 is a transcriptional activator that represses plant basal defense. Molecular plant, 2008, 1(3): 459-470. |
| 4 | *ATWRKY8* | 834678 | *AT5G46350* | Response to salt tolerant | Hu Y, Chen L, Wang H, et al. A rabidopsis transcription factor WRKY 8 functions antagonistically with its interacting partner VQ 9 to modulate salinity stress tolerance. The Plant Journal, 2013, 74(5): 730-745. |
| 5 | *ATWRKY23* | 819339 | *AT2G47260* |  |  |
| 6 | *ATWRKY57* | 843262 | *AT1G69310* | Response to salt tolerant | Jiang Y, Liang G, Yu D. Activated expression of WRKY57 confers drought tolerance in Arabidopsis. Molecular plant, 2012, 5(6): 1375-1388. |
| 7 | *ATWRKY68* | 825407 | *AT3G62340* |  |  |
| 8 | *ATWRKY3* | 814863 | *AT2G03340* |  |  |
| 9 | *ATWRKY4* | 837956 | *AT1G13960* |  |  |
| 10 | *ATWRKY26* | 830601 | *AT5G07100* | Response to heat tolerant | Li S, Fu Q, Chen L, et al. Arabidopsis thaliana WRKY25, WRKY26, and WRKY33 coordinate induction of plant thermotolerance. Planta, 2011, 233(6): 1237-1252. |
| 11 | *ATWRKY13* | 830096 | *AT4G39410* | Regulate flowering time | Li W, Wang H, Yu D. Arabidopsis WRKY transcription factors WRKY12 and WRKY13 oppositely regulate flowering under short-day conditions. Molecular plant, 2016, 9(11): 1492-1503. |
| 12 | *ATWRKY43* | 819220 | *AT2G46130* |  |  |
| 13 | *ATWRKY56* | 842703 | *AT1G64000* |  |  |
| 14 | *ATWRKY50* | 832686 | *AT5G26170* |  |  |
| 15 | *ATWRKY24* | 834159 | *AT5G41570* |  |  |
| 16 | *ATWRKY58* | 821213 | *AT3G01080* |  |  |
| 17 | *ATWRKY45* | 821270 | *AT3G01970* | Response to phosphate starvation | Wang H, Xu Q, Kong Y H, et al. Arabidopsis WRKY45 transcription factor activates PHT1; 1 expression in response to phosphate starvation. Plant physiology, 2014: pp. 113.235077. |
| 18 | *OsWRKY72* | 4350545 | *OSNPB_110490900* |  |  |
| 19 | *OsWRKY16* | 4326856 | *OSNPB_010665500* |  |  |
| 20 | *OsWRKY11* | 4326690 | *OSNPB_010626400* |  |  |
| 21 | *OsWRKY29* | 4342232 | *OSNPB_070111400* |  |  |
| 22 | *OsWRKY3* | 4334170 | *OSNPB_030758000* |  |  |
| 23 | *OsWRKY49* | 9268095 | *OSNPB_050565900* |  |  |
| 24 | *OsWRKY8* | 4339739 | *OSNPB_050583000* |  |  |
| 25 | *OsWRKY7* | 4339451 | *OSNPB_050537100* |  |  |
| 26 | *OsWRKY23* | 4324161 | *OSNPB_010734000* |  |  |
| 27 | *OsWRKY26* | 4325945 | *OSNPB_010714800* |  |  |
| 28 | *OsWRKY77* | 4327651 | *OSNPB_010584900* | Involved in disease resistance | Lan A, Huang J, Zhao W, et al. A salicylic acid‐induced rice (Oryza sativa L.) transcription factor OsWRKY77 is involved in disease resistance of Arabidopsis thaliana. Plant Biology, 2013, 15(3): 452-461. |
| 29 | *OsWRKY72* | 4350545 | *OSNPB_110490900* |  |  |
| 30 | *OsWRKY81* | 4333225 | *OSNPB_030444900* |  |  |
| 31 | *OsWRKY88* | 4343795 | *OSNPB_070596900* |  |  |
| 32 | *OsWRKY10* | 4325658 | *OSNPB_010186000* |  |  |
| 33 | *OsWRKY87* | 4343736 | *OSNPB_070583700* |  |  |
| 34 | *OsWRKY102* | 4325330 | *OSNPB_010182700* |  |  |
| 35 | *OsWRKY96* | 4352301 | *OSNPB_120507300* |  |  |
| 36 | *GmWRKY1* | Glyma.01G043300 | *GmWRKY3* | Glyma01g05050 |  |
| 37 | *GmWRKY2* | Glyma.01G053800 | *GmWRKY9* | Glyma01g06550 |  |
| 38 | *GmWRKY3* | Glyma.01G056800 | *GmWRKY28* | Glyma01g06870 |  |
| 39 | *GmWRKY4* | Glyma.01G128100 | *GmWRKY5* | Glyma01g31921 |  |
| 40 | *GmWRKY5* | Glyma.01G189100 | *GmWRKY35* | Glyma01g39600 |  |
| 41 | *GmWRKY6* | Glyma.01G222300 | *GmWRKY65* | Glyma01g43130 |  |
| 42 | *GmWRKY7* | Glyma.01G224800 | *GmWRKY12* | Glyma01g43420 |  |
| 43 | *GmWRKY8* | Glyma.02G007500 | *GmWRKY66* | Glyma02g01031 |  |
| 44 | *GmWRKY9* | Glyma.02G010900 | *GmWRKY67* | Glyma02g01420 |  |
| 45 | *GmWRKY10* | Glyma.02G020300 | *GmWRKY68* | Glyma02g02430 |  |
| 46 | *GmWRKY11* | Glyma.02G112100 | *GmWRKY69* | Glyma02g12490 |  |
| 47 | *GmWRKY12* | Glyma.02G115200 | *GmWRKY32* | Glyma02g12830 |  |
| 48 | *GmWRKY13* | Glyma.02G141000 | *GmWRKY22* | Glyma02g15920 |  |
| 49 | *GmWRKY14* | Glyma.02G203800 | *GmWRKY70* | Glyma02g36510 |  |
| 50 | *GmWRKY15* | Glyma.02G232600 | *GmWRKY39* | Glyma02g39870 |  |
| 51 | *GmWRKY16* | Glyma.02G285900 | *GmWRKY71* | Glyma02g45530 |  |
| 52 | *GmWRKY17* | Glyma.02G293400 | *GmWRKY72* | Glyma02g46280 |  |
| 53 | *GmWRKY18* | Glyma.02G297400 | *GmWRKY73* | Glyma02g46690 |  |
| 54 | *GmWRKY19* | Glyma.02G306300 | *GmWRKY74* | Glyma02g47650 |  |
| 55 | *GmWRKY20* | Glyma.03G002300 | *GmWRKY75* | Glyma03g00460 |  |
| 56 | *GmWRKY21* | Glyma.03G042700 | *GmWRKY76* | Glyma03g05220 |  |
| 57 | *GmWRKY22* | Glyma.03G109100 | *GmWRKY77* | Glyma03g25770 |  |
| 58 | *GmWRKY23* | Glyma.03G159700 | *GmWRKY15* | Glyma03g31630 |  |
| 59 | *GmWRKY24* | Glyma.03G176600 | *GmWRKY29* | Glyma03g33376 |  |
| 60 | *GmWRKY25* | Glyma.03G220100 | *GmWRKY41* | Glyma03g37870 |  |
| 61 | *GmWRKY26* | Glyma.03G220800 | *GmWRKY51* | Glyma03g37940 |  |
| 62 | *GmWRKY27* | Glyma.03G224700 | *GmWRKY78* | Glyma03g38360 |  |
| 63 | *GmWRKY28* | Glyma.03G256700 | *GmWRKY43* | Glyma03g41750 |  |
| 64 | *GmWRKY29* | Glyma.04G054200 | *GmWRKY79* | Glyma04g05700 |  |
| 65 | *GmWRKY30* | Glyma.04G061300 | *GmWRKY80* | Glyma04g06470 |  |
| 66 | *GmWRKY31* | Glyma.04G061400 | *GmWRKY81* | Glyma04g06495 |  |
| 67 | *GmWRKY32* | Glyma.04G076200 | *GmWRKY50* | Glyma04g08060 |  |
| 68 | *GmWRKY33* | Glyma.04G115500 | *GmWRKY82* | Glyma04g12830 |  |
| 69 | *GmWRKY34* | Glyma.04G173500 | *GmWRKY83* | Glyma04g34220 |  |
| 70 | *GmWRKY35* | Glyma.04G218400 | *GmWRKY45* | Glyma04g39621 |  |
| 71 | *GmWRKY36* | Glyma.04G218700 | *GmWRKY21* | Glyma04g39650 | Zhou Q Y, Tian A G, Zou H F, et al. Soybean WRKY‐type transcription factor genes, GmWRKY13, GmWRKY21, and GmWRKY54, confer differential tolerance to abiotic stresses in transgenic Arabidopsis plants [J]. Plant biotechnology journal, 2008, 6(5): 486-503. |
| 72 | *GmWRKY37* | Glyma.04G223200 | *GmWRKY84* | Glyma04g40121 |  |
| 73 | *GmWRKY38* | Glyma.04G223300 | *GmWRKY58* | Glyma04g40130 | Yang Y, Chi Y, Wang Z, et al. Functional analysis of structurally related soybean GmWRKY58 and GmWRKY76 in plant growth and development [J]. Journal of experimental botany, 2016, 67(15): 4727-4742. |
| 74 | *GmWRKY39* | Glyma.04G238300 | *GmWRKY85* | Glyma04g41701 |  |
| 75 | *GmWRKY40* | Glyma.05G029000 | *GmWRKY86* | Glyma05g01285 |  |
| 76 | *GmWRKY41* | Glyma.05G096500 | *GmWRKY11* | Glyma05g20710 |  |
| 77 | *GmWRKY42* | Glyma.05G123000 | *GmWRKY87* | Glyma05g25270 |  |
| 78 | *GmWRKY43* | Glyma.05G123600 | *GmWRKY88* | Glyma05g25331 |  |
| 79 | *GmWRKY44* | Glyma.05G127600 | *GmWRKY89* | Glyma05g25770 |  |
| 80 | *GmWRKY45* | Glyma.05G160800 | *GmWRKY90* | Glyma05g29310 |  |
| 81 | *GmWRKY46* | Glyma.05G165800 | *GmWRKY91* | Glyma05g29921 |  |
| 82 | *GmWRKY47* | Glyma.05G184500 | *GmWRKY92* | Glyma05g31800 |  |
| 83 | *GmWRKY48* | Glyma.05G185400 | *GmWRKY93* | Glyma05g31910 |  |
| 84 | *GmWRKY49* | Glyma.05G203900 |  |  |  |
| 85 | *GmWRKY129* | Glyma.14G028900 |  |  |  |

A comparison of *GmWRKY* genes identified in current study with those of previous study ([Bencke-Malato et al, 2014).](#_ENREF_1)
