# Supplementary material for: GmWRKY16 Enhances Drought and Salt Tolerance Through an ABA-Mediated Pathway in Arabidopsis thaliana
Source: Front Plant Sci. 2019 Jan 21;9:1979. doi: 10.3389/fpls.2018.01979 (PMC6357947; doi:10.3389/fpls.2018.01979)
Supplement: Supplementary file 3 [file Image_1.pdf]

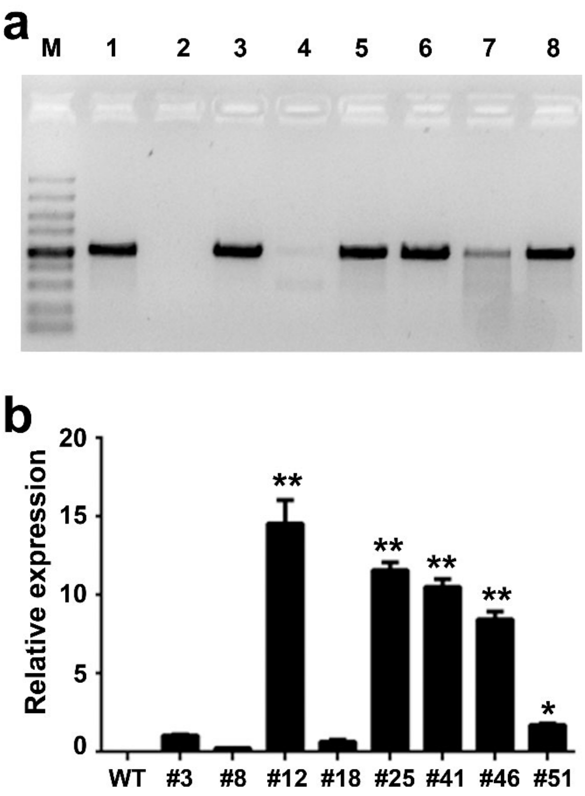

Fig.S1 Molecular identification of *GmWRKY16* transgenic Arabidopsis lines

a. PCR identification of *GmWRKY16* transgenic Arabidopsis plants. **b**. qRT-PCR identification of *GmWRKY16* transgenic Arabidopsis lines. The three-week-old seedlings were used to perform the molecular identification of *GmWRKY16* transgenic Arabidopsis plant and lines. M: DNA marker; Lane 1-8: PCR products with different DNA templates set as GmWRKY16-pLB plasmid for lane 1, ddH_2O for lane 2, genomic DNA from randomly selected *GmWRKY16* transgenic Arabidopsis plants for lane 3-8. WT: wild type; #3, 8, 12, 18, 25, 41, 46, and 51: *GmWRKY16* transgenic Arabidopsis lines of T_3 generation.
