# Supplementary material for: GmWRKY16 Enhances Drought and Salt Tolerance Through an ABA-Mediated Pathway in Arabidopsis thaliana
Source: Front Plant Sci. 2019 Jan 21;9:1979. doi: 10.3389/fpls.2018.01979 (PMC6357947; doi:10.3389/fpls.2018.01979)
Supplement: Supplementary file 4 [file Image_2.pdf]

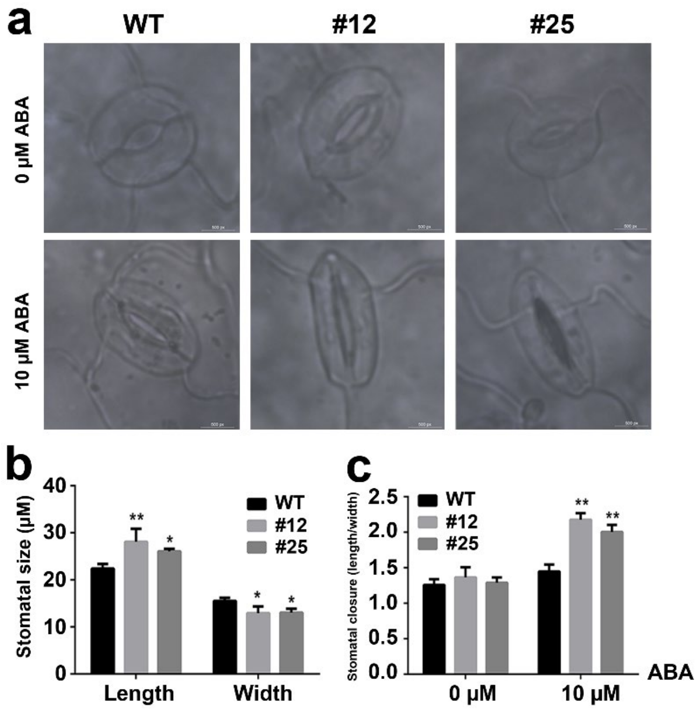

Fig.S2 Stomatal aperture of *GmWRKY16* transgenic Arabidopsis lines response to ABA

a. The phenotype of stomatal aperture of *GmWRKY16* transgenic Arabidopsis lines. Seed germination under ABA treatment. **b-c**. The size and closure of stoma. The rosette leaves from 4-week-old seedlings were broken into a transparent epidermis strip using a grinding machine. The epidermal strips were immersed in a guard cell buffer before they were incubated under light (150 μmol m^{-2} s^{-1}) for 2 h. The ground blades were then transferred to a solution containing same buffer with 0 and 10 μM ABA. After incubation for 2 h under white light, 30 stomatal apertures in each epidermal peel were measured and analyzed as described previously (Seo et al., 2012). 60 stomata cells from each sample were used to determinate the status of stomatal aperture in triple replicates. Data are means ± SD. Three independent biological experiments were performed to investigate stomatal aperture of WT and GmWRKY16 transgenic lines under ABA stress. WT: wild type; #12, 25: *GmWRKY16* Arabidopsis transgenic lines of T_3 generations.
